# Comparing Docetaxel Plus Cisplatin with Paclitaxel Plus Carboplatin in Chemotherapy-Naïve Patients with Advanced Non-Small-Cell Lung Cancer: a Single Institute Study

**Published:** 2014

**Authors:** Kian Khodadad, Adnan Khosravi, Zahra Esfahani-Monfared, Shirin Karimi, Sharare Seifi

**Affiliations:** a *Chronic Respiratory Diseases Research Center, National Research Institute of Tuberculosis and Lung Diseases (NRITLD), Shahid Beheshti University of Medical Sciences, Tehran, Iran.*; b *Tobacco Prevention and Control Research Center, National Research Institute of Tuberculosis and Lung Diseases (NRITLD), Shahid Beheshti University of Medical Sciences ,Tehran, Iran. *; c *Mycobacteriology Research Center, National Research Institute of Tuberculosis and Lung Diseases (NRITLD), Shahid Beheshti University of Medical Sciences, Tehran, Iran.*

**Keywords:** Docetaxel, Paclitaxel, Non-small cell, Lung cancer

## Abstract

The backbone of treatment in advanced non-small cell lung cancer is platinum-based doublet chemotherapy. We intended to compare the effectiveness of two commonly used regimens in real world practice.

This single institute, parallel comparative post marketing study included 100 patients with chemo-naïve advanced (stage IIIB, IV) non-small cell lung cancer and Eastern Cooperative Oncology Group performance status of 0 to 2. They were randomly assigned by stratified blocks to receive Docetaxel/Cisplatin (DC, n=50) on day 1 or Paclitaxel/Carboplatin AUC 5 (PC, n=50) on day 1, every 3 weeks for up to six cycles. Primary end point was progression free survival (PFS); secondary end points were objective response rate, overall survival (OS) and toxicity. The administered dosage could be modified according to clinician’s discretion for each individual patient.

PFS was similar between DC and PC arms (4.5 ± 0.3 *v* 4.6 ± 1.8 months, respectively; HR= 1.337; 95% CI: 0.874 to 2.046, P = 0.181). Although median overall survival for DC arm was longer (17.2 ± 4.4 m) than PC arm (10.6 ± 0.7 m) but was not statistically significant (P = 0.300). The 1-year survival rates were in favor of DC arm (53.1% *v* 37.9%). Objective response rates were similar in both groups. In our study, hematologic toxicity and neuropathy were more frequent in DC and PC arms, respectively.

In our study two commonly used regimens of DC and PC showed statistically similar outcomes in terms of PFS and OS, albeit numerically results of OS and 1-year survival were in favor of DC arm.

## Introduction

Lung cancer has remained as a worldwide public health threat with over 1.3 million new cases every year (1). It is the second most common cancer for both men and women (2). Eighty percent of lung cancer are non**-**small-cell (NSCLC) and at the time of diagnosis most of them are locally advanced (inoperable stage IIIB) or metastatic (stage IV) (3). In advanced NSCLC doublet combinations of platinums (4)(carboplatin or cisplatin) with taxanes (docetaxel or paclitaxel), gemcitabine or vinorelbine are backbone of standard treatment (5). Although these regimens have different toxicity profiles but their efficacies are similar in head-to-head phase III trials (6-9). Meanwhile, the addition of bevacizumab, a monoclonal antibody against vascular endothelial growth factor, to paclitaxel/carboplatin has led to improved response rate and survival benefit, although in expense of increased risk of particular treatment-related side morbidities and mortalities (10).

In this regard, the selection of platinum-doublet chemotherapy regimen is based on clinician's experience, patient’s comorbid disease, as well as drug anticipated toxicity and also, flexibility in the administration schedule. The aim of this study was to determine the comparison of two standard first-line chemotherapy regimens in advanced NSCLC: docetaxel/cisplatin versus paclitaxel/carboplatin– in terms of survival and toxicity profile in a comparative post marketing study. 

## Experimental

*Methods*

The chemotherapy-naïve patients with histologically or cytologically confirmed NSCLC, at stage Eastern Cooperative Oncology Group performance status (PS) 0-2 and stages IIIB, wet IIIB and IV (by AJCC, 6^th^ edition) were enrolled in this study. Other eligibility criteria included the following: age ≥ 18 years old, at least one unidimensionally measurable or assessable disease, adequate bone marrow reserve, serum creatinine less than or equal to 1.5 mg/dL or a calculated creatinine clearance greater than or equal to 60 mL/min, bilirubin level less than or equal to 2.0 mg/dL, AST less than or equal to twice the institutional upper limits of normal, or less than or equal to four times the institutional upper limits of normal if the patient had liver metastasis. Neither of patients had prior chemotherapy, biologic therapy or radiotherapy less than 14 days ago. 

**Figure 1:**
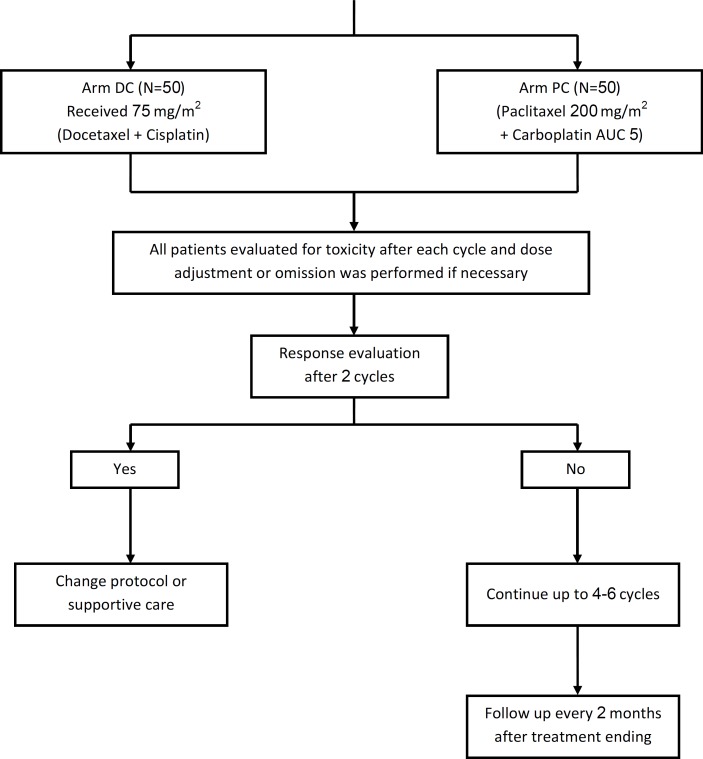
Diagram of the study; clinical trial flow chart. A total of 100 patients received study treatment consisting of at least one dose of Docetaxel / Cisplatin (DC; n = 50) or Paclitaxel/Carboplatin (PC; n = 50).

Eligible patients randomly assigned to receive either docetaxel plus cisplatin, both 75 mg/m^2 ^on day 1 (DC arm) or paclitaxel 200 mg/m^2 ^plus carboplatin AUC 5 (PC arm) on day 1 every 3 weeks. Clinical trial flow chart is showed in Figure 1. Notably, in this *real world* study the dosage of cytotoxic agents was permitted to be modified by clinicians’ discretion based on patient’s age, alteration on PS, or adverse events in the course of treatment for each individual case. In the absence of progressive disease or intolerable toxicity, the patients were treated for a minimum of four cycles. Patients who achieved a complete or partial response could receive two additional cycles of therapy, for a maximum of 6 cycles.

During treatment all patients had a complete blood cell (CBC) count, one week after each chemotherapy cycle. Dose modification and concomitant G-CSF were allowed during treatment course according to the encountered toxicity. 

*Treatment assessments*

Patients were evaluated after every cycle for any response based on physical exam and chest X-ray. Chest CT scan was requested after every 2 cycles and/or at the termination of protocol. Disease assessment was performed according to “Response Evaluation Criteria in Solid Tumors”(11). Objective response was consisted with CR+PR response. Combination of CR, PR and SD was defined as disease control. Patients who had received at least two cycles of chemotherapy were considered assessable for tumor response. Toxicity grading was performed in accordance with the National Cancer Institute common toxicity criteria, version 2.0 (12). All patients who received at least one cycle of chemotherapy were considered assessable for safety. 

Efficacy analysis included progression free survival (PFS) as primary end point and median overall survival (OS), objective response rate and toxicity as secondary end points. 

Criteria for withdrawal from study were unacceptable toxicity as determined by the treating physician in consultation with the study coordinator, a delay in treatment greater than 2 weeks, requirement for palliative radiotherapy, or patient refusal.

*Statistical analysis*

The sample size was determined using a significance level of 5% for alpha to test the hypothesis that two treatment arms were equal in term of response rat, PFS and toxicity. Finally, enrolled a total of 100 patients when probable losses to follow up were taken in to account. 

For testing the differences in categorical variables between two arms, the chi-square test or Fisher’s exact test was used. The difference in quantitative variables of two groups was compared using the Student's t-test or non-parametric Mann-Whitney test. Kaplan Meier's survival estimates and curves were obtained and the log-rank test was used to assess the significance of differences of the OS and PFS between two study groups. PFS was calculated from date of registration to date of progression or death. Survival time listed from date of registration. COX-PH regression model was used to estimate hazard ratios and their 95% CIs (confidence intervals).

For all statistical tests, the 5% level was used as cutoff for statistical significance. All analysis was performed using SPSS version 16.

## Results

From August 2007 to January 2010, a total of 100 eligible patients were randomly assigned in one of two arms. Patient demographics and other characteristics in both groups at baseline are showed in Table 1. Female/male ratio was 1.6. There was no significant difference in any of the characteristics listed between the two groups including gender, PS, stage and histologic subtype. Mean of total administered cycles in DC and PC arms were 3.5 ± 1.5 and 3.8 ± 1.8, respectively (P = 0.35). Median duration of treatment by DC and PC protocol were 2.61 m and 2.43 m, respectively (P = 0.914). 

*Response and survival*

**Figure 2:**
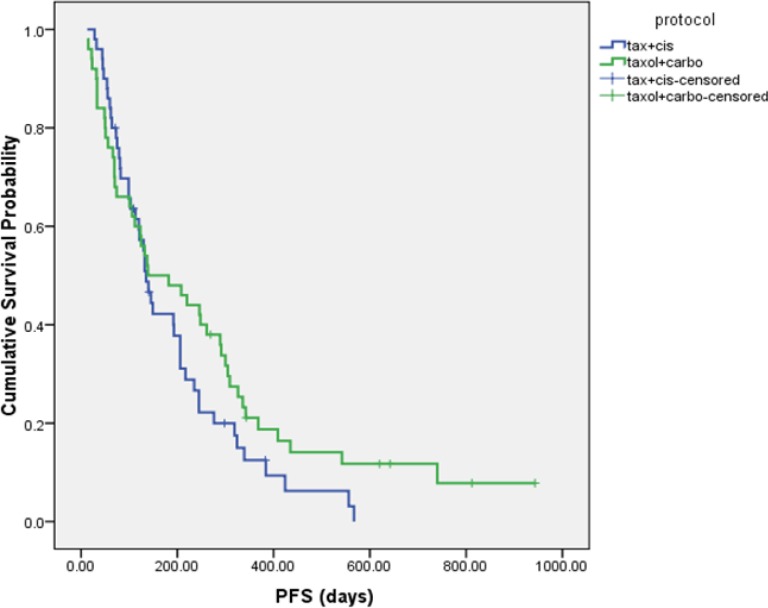
Kaplan-Meier progression-free survival (PFS) curve in patients in DC and PC arms.

**Figure 3:**
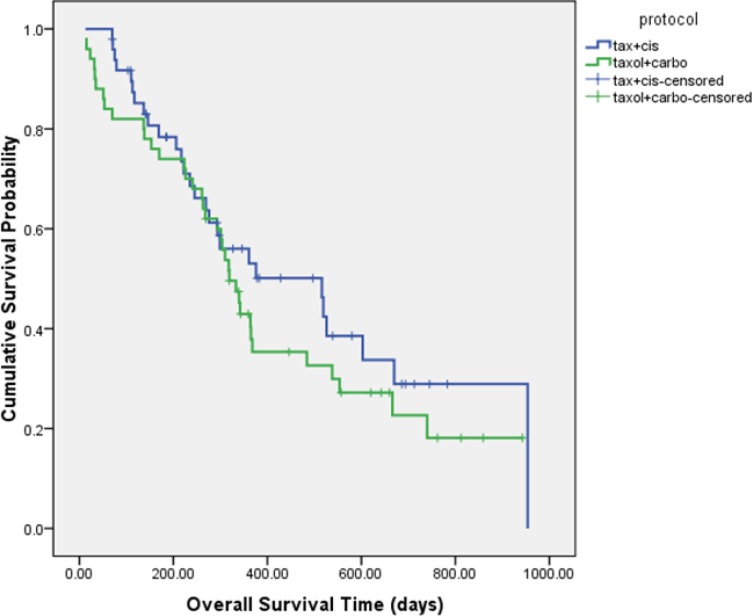
Kaplan-Meier overall survival curve in patients in DC and PC arms

The median PFS was 4.5 ± 0.3 and 4.6 ± 1.8 months in DC and PC arms, respectively (HR= 1.337, 95% CI: 0.874 to 2.046, P = 0.181 (Figure 2). Although median overall survival for DC arm was numerically longer (17.2 ± 4.4 m) than PC arm (10.63 ± 0.7 m) but it was not statistically significant (HR = 0.766, 95% CI: 0.462 to 1.270, P = 0.301). Figure 3 shows the Kaplan-Meier curve for overall survival. Survival rate at 12 months were 53.1% and 37.9% for DC and PC arms, respectively. Albeit objective response rates were higher in PC arm but it was statistically non-significant compare to DC arm (PC = 32.7%, DC = 24%, P = 0.339). Disease control rates were similar in both arms (DC = 80%, PC = 73.7%, P = 0.442) (Table 2).

**Table 1:** Baseline clinicopathologic characteristics for randomly assigned patients

**Variables**	**Arm DC No. (%)**	**Arm PC No. (%)**	**Total No. (%)**	**p-value**
**Sex**				0.410
Male	21(42%)	17(34%)	38(38%)	
Female	29(58%)	33(66%)	62(62%)	
**Age**				0.638
(Mean±SD)	51.5±10.8	50.6±8.5	51.1±9.7	
**Stage of disease**				0.156
IIIB, dry	5(10%)	9(18%)	14(14%)	
IIIB, wet	8(16%)	6(12%)	14(14%)	
IV	25(50%)	35(70%)	72(72%)	
**ECOG performance status**				0.156
0	0(0)	0(0)	0(0)	
1	25(50%)	17(34%)	42(42%)	
2	25(50%)	33(66%)	25(50%)	
**Histologic subtype**				0.769
Adenocarcinoma	27(54%)	24(48%)	51(51%)	
Squamous cell carcinoma	8(16%)	10(20%)	18(18%)	
Undetermined NSCLC	14(28%)	16(32%)	30(30%)	
Large-cell carcinoma	1 (2%)	0(0%)	1(1%)	

Median follow up was 10.03 months and 5 cases were lost on follow-up. At the time of analysis 22 (44%) patients in arm DC and 24 (36%) patients in arm PC were alive (P = 0.096). Mean of chemotherapy cycles in DC arm was 3.5 cycles compare to 3.8 in PC arm. Post protocol salvage chemotherapy was administered in 22 (45.8%) and 25 (53.2%) patients in arm DC and PC, respectively (P = 0.473). Eighteen patients in arm DC received radiotherapy versus 8 patients in arm PC (P = 0.023) although the intention of radiation (complementary to chemotherapy *v* palliative) was similar in two arms (P = 0.628).

**Table 2:** Responses to treatment in both arms

**Objective response**	**Arm DC**	**Arm PC**	**P-Value**
CR+PR	12(24%)	16(32.7%)	0.339
CR+PR+SD	40(80%)	36(73.7%)	0.442
Progression	10(20%)	8(16.3%)	0.636
NA	0(0%)	5(10.2%)	0.027

All patients in both arms were assessable for toxicity. Important grade 2 and 3 toxicities have showed in Table 3. Neuropathy was more common in PC arm. In contrast, as expected, nausea and vomiting were significantly more frequent in DC arm. Albeit, grade 2 and 3 leukopenia occurred more frequently in patients who received the docetaxel regimen compare with patients who received the paclitaxel regimen (12 *v* 7 patients), but this was not statistically significant. No patient developed grade 4 febrile neutropenia, but five patients experienced grade 3 febrile neutropenia; four in DC arm and one in PC arm. Grade 2 anemia was more common in arm PC (P = 0.059). Furthermore, one patient on DC arm developed grade 3 cutaneous reaction after fifth cycle of chemotherapy. No patient developed significant renal toxicity in this study.

Toxicity concerns also dictated the dose of administered cytotoxic agents. In review of total administrated dosage of these agents demonstrated that during the planned chemotherapy cycles, the patients had received 80.8%, 82.3%, 83.06% and 87.3% of optimal doses of docetaxel, paclitaxel, cisplatin and carboplatin, respectively. 

**Table 3:** Grade 2 and 3 adverse events in both arms

**Adverse Event**	**Grade 2**		**p-value**	**Grade 3**		**p-value**
	**Arm DC**	**Arm PC**		**Arm DC**	**Arm PC**	
Neuropathy	4(8%)	11(22%)	0.050	1(8%)	0(8%)	>0.999
Nausea/Vomiting	14(28%)	4(8%)	0.009	10(20%)	0(0%)	0.001
Diarrhea	6(12%)	4(8%)	0.505	0(0%)	2(4%)	0.495
Leukopenia	8(16%)	3(6%)	0.110	4(8%)	4(8%)	>0.999
Anemia	1(2%)	7(14%)	0.509	1(2%)	0(0%)	>0.999
Neutropenia	5(10%)	5(10%)	>0.999	2(4%)	1(2%)	>0.999
Thrombocytopenia	0(0%)	2(8%)	0.495	0(0%)	0(0%)	-

## Discussion

Few standard chemotherapy protocols have been approved for treatment of advanced NSCLC. Nevertheless, in various countries the choice of protocol should ultimately take into consideration based on a number of factors. These factors could be classified as ministerial (availability and approval of brand and/or generic cytotoxic agents, and their coverage by insurances), institutional (patients load and turnover, personnel shortages, cost of administration and hospitalization), as well as patient factors (comorbid diseases, performance status, out of pocket cost, patients discretion on anticipated adverse events secondary to chemotherapy protocol, convenience of protocol schedules) and physician discretion (familiarity with protocol, management of its adverse effects, and consideration of aforementioned factors all together). 

Platinum-based regimens of DC and PC are two protocols that are used so frequently in our institution in this setting. The main reason is their administration schedule (every 3 weeks). These regimens are more convenient for our thoracic oncology ward which is overloaded with referral patients from all cross country and suffering shortage of personnel (including physicians and nurses) and limited budget. 

This study was conducted to evaluate the *effectiveness* of these currently used regimens in *real world* practice. In our day-to-day practice for different reasons we cannot implement the standard protocols in terms of dosage. In other words, somehow we might sacrifice some efficacy in favor of less severe manageable adverse events. Although this approach with suboptimal dosage could jeopardize treatment outcome but attenuate the morbidity and mortality of treatment in patients' population whose treatment is a sort of palliative care at best. The importance of this issue is more prominent when we are talking of patients treatment in developing countries, where the more specialized and tertiary centers as expertise medical staff and physicians are not available in every part of country and for each adverse events they could not access to their main chemotherapy clinic. For these reasons despite the defined dosage for cytotoxic agents the clinicians in this study were permitted to modify these dosages according to their patient’s age, PS, comorbid condition, or their clinical judgment experience. In our study the mean of administrated dosage of cytotoxic agents have been around 80% of the proposed standard dosage. Consequently, most probably in case of full dose administration of cytotoxic agents we could achieve better results in expense of more frequent and severe adverse events. In this study we did not intend to compare the efficacy of two different taxanes or platinum compounds with each other but comparing two different combination chemotherapy regimens.

In this regard, our study demonstrated that the doublet regimens of docetaxel plus cisplatin and paclitaxel plus carboplatin produced equivalent response rates and survival in treatment of advanced NSCLC. This is when both arms are relatively identical in terms of patients’ age, gender ratio, histologic subtypes and PS. Of note, despite identical PFS, numerically the MOS and 1-year survival were improved in favor of DC arm, although these differences were not statistically significant. This could be due to cisplatin in DC arm compare to carboplatin in PC arm, although there is a debate over cisplatin to be more efficient compare to carboplatin in doublet regimens (13). Furthermore, presumably the more radiation administration in DC arm may interpret this finding.

Albeit the effective of these regimens are identical but their toxicity profiles differ. There was more nausea in DC arm and more neuropathy in PC arm as was expected secondary to cisplatin and paclitaxel use, respectively. 

In conclusion, newer cytotoxic agents plus platinum and new therapies (14) have led to a modest increase in survival for patients with advanced lung cancer, and their continued evaluation is important. This *real world* study demonstrated that two standard regimens of DC and PC are comparable in terms of RR, PFS and MOS. However, their different toxicity profile may dictate the chosen regimen. We propose that the *effectiveness* of different current standard chemotherapy regimens (+/- biologic agents) be evaluated and compared in the *real world* setting. As the result of this sort of studies could be different from the results reported from highly selected patient population in phase II or III clinical trials. In this regard more emphasis is warranted to cost/effectiveness of these regimens. The importance of these issues will be more evident when we consider the diversity of treatment facilities, trained and expert personnel, financial constrains or even patients’ culture as confounding factors that could have a great impact on the selection of systemic treatment in patients with advanced NSCLC.

This study has been partially supported by Sanofi-Aventis.
